# Correction: Abcg2a is the functional homolog of human ABCG2 expressed at the zebrafish blood–brain barrier

**DOI:** 10.1186/s12987-024-00606-9

**Published:** 2025-01-03

**Authors:** Joanna R. Thomas, William J. E. Frye, Robert W. Robey, Andrew C. Warner, Donna Butcher, Jennifer L. Matta, Tamara C. Morgan, Elijah F. Edmondson, Paula B. Salazar, Suresh V. Ambudkar, Michael M. Gottesman

**Affiliations:** 1 Laboratory of Cell Biology, Center for Cancer Research, National Cancer Institute, National Institutes of Health, 37 Convent Drive, Room 2108, Bethesda, MD 20892 USA; 2 Molecular Histopathology Laboratory, Frederick National Laboratory for Cancer Research, Frederick, MD USA

**Correction: Fluids Barriers CNS21, 27 (2024)**

10.1186/s12987-024-00529-5

Following publication of this article [1], it was brought to our attention that Supplementary Fig. 3 was published incorrectly. Instead of the intended figure and legend, the published version mistakenly included a duplicate of Fig. 7 and its legend.

The correct and incorrect version of Supplementary Fig. 3 along with its legend are provided below:

**Correct version**

**Additional file 3: Figure S3:**
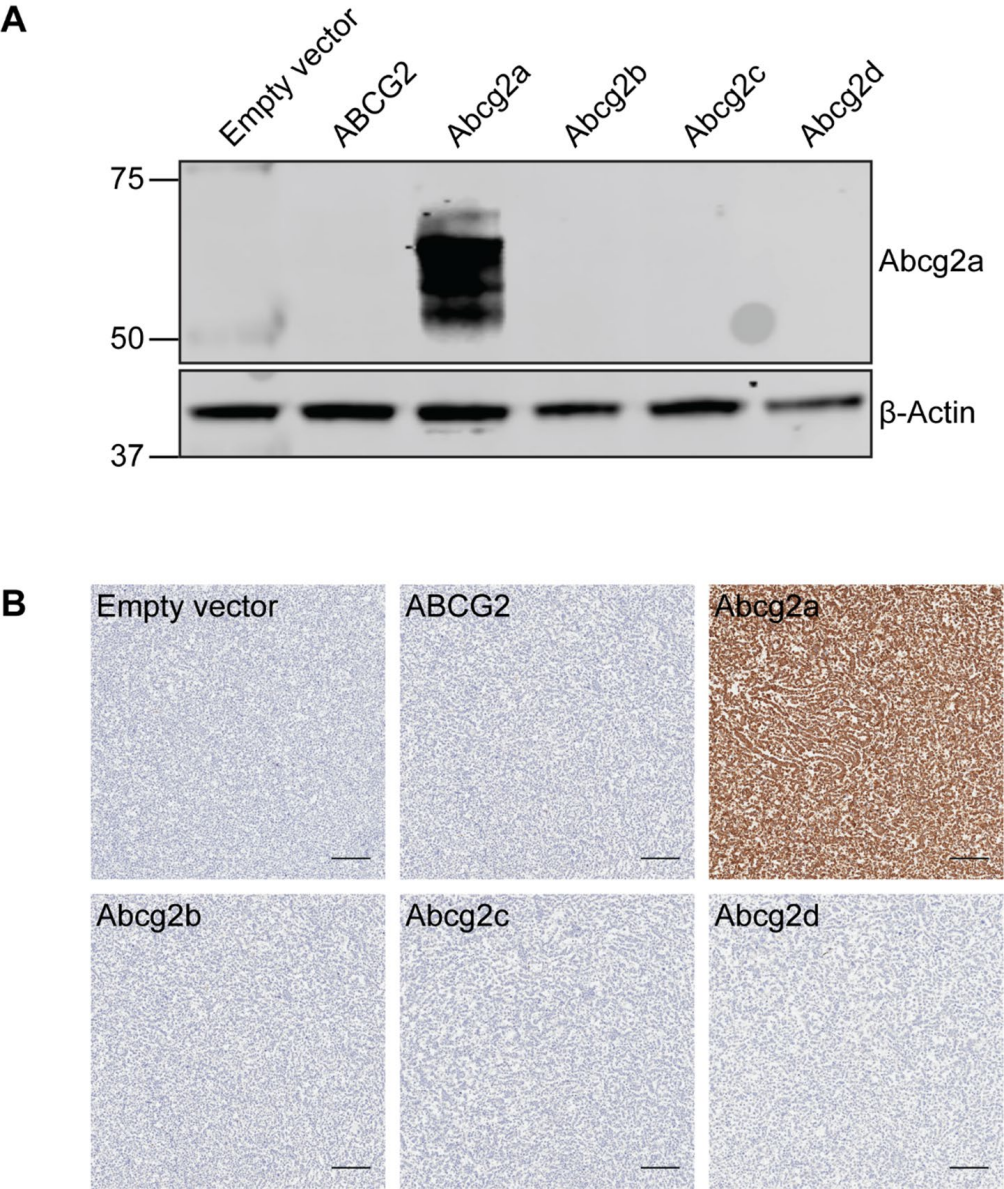
Abcg2a antibody validation. **(A)** Immunoblot of total cell lysates and **(B)** immunohistochemistry of pellets of transfected HEK-293 cells expressing an empty vector, ABCG2, Abcg2a, Abcg2b, Abcg2c, or Abcg2d. Positive signal is only observed in Abcg2a-expressing cells. Scale bar = 100 μm

**Incorrect version**

**Additional file 3: Figure S3:**
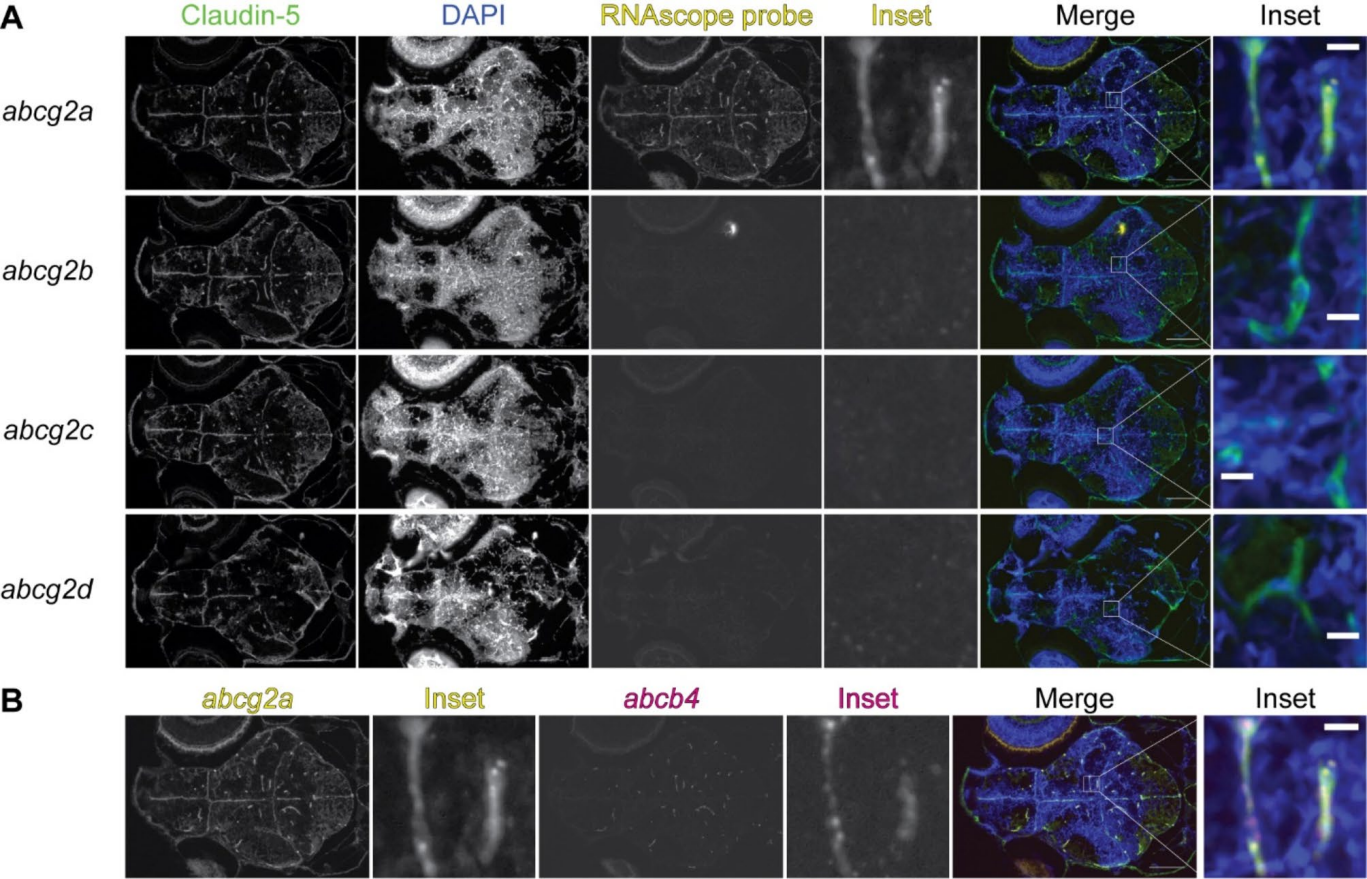
abcg2a and abcb4 are expressed in claudin-5 positive 5 dpf larval brain vasculature. **A** Paraffin-embedded 5 dpf larval zebrafish sections were probed with RNAscope probes (yellow) to detect abcg2a-d mRNA, an antibody against claudin-5 (green) and DAPI (blue). **B** Co-staining of the abcg2a probed section with an abcb4 RNAscope probe. Scale bar = 100 μm, inset scale bar = 10 μm

The original article has been corrected.
