# Supplementary figures and images for: Abcg2a is the functional homolog of human ABCG2 expressed at the zebrafish blood–brain barrier
Source: Fluids Barriers CNS. 2024 Mar 15;21:27. doi: 10.1186/s12987-024-00529-5 (PMC10941402; doi:10.1186/s12987-024-00529-5)

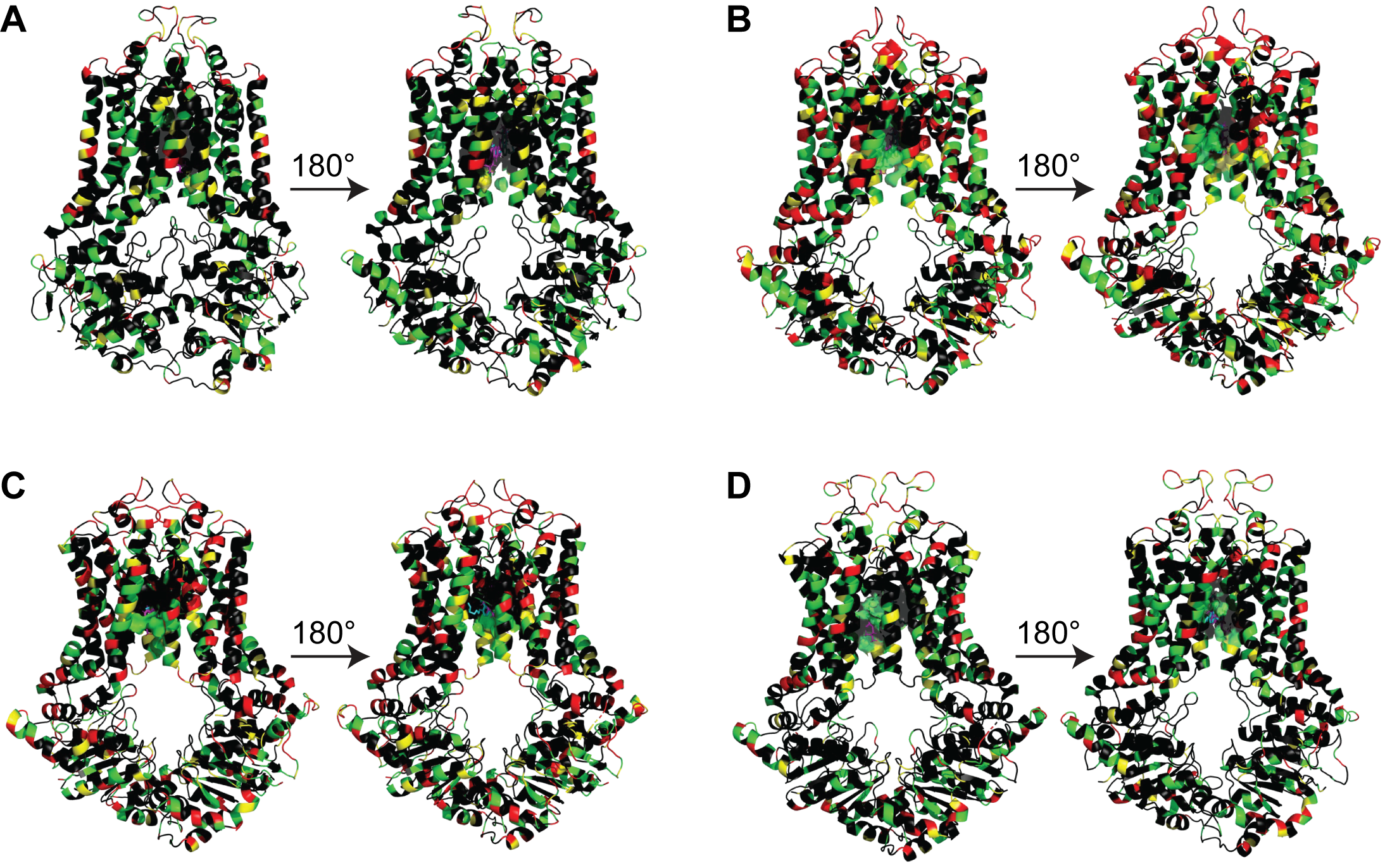

Supplement: Supplementary file 1 — Additional file 1: Figure S1. 3-D homology modeling of sequence similarity of zebrafish A Abcg2a, B Abcg2b, C Abcg2c, and D Abcg2d. Predicted structures of zebrafish Abcg2a-d paralogs were aligned to the cryo-EM structure of human ABCG2 (PDB IDs: 7NEZ and 6VXI for topotecan and mitoxantrone bound structures, respectively). Structures are presented in cartoon mode with a 180° rotated view, and the predicted drug binding pocket is presented in surface mode. Amino acids are color coded black (fully conserved), green (conservative substitution), yellow (semi-conservative substitution), and red (non-conservative substitution) based on similarity to ABCG2. Clustal amino acid sequence comparisons comparing Abcg2 paralogs with ABCG2 calculated percent identities of 63.26%, 47.28%, 47.92%, and 61.31% for Abcg2a, Abcg2b, Abcg2c, and Abcg2d, respectively. [file 12987_2024_529_MOESM1_ESM.tif]

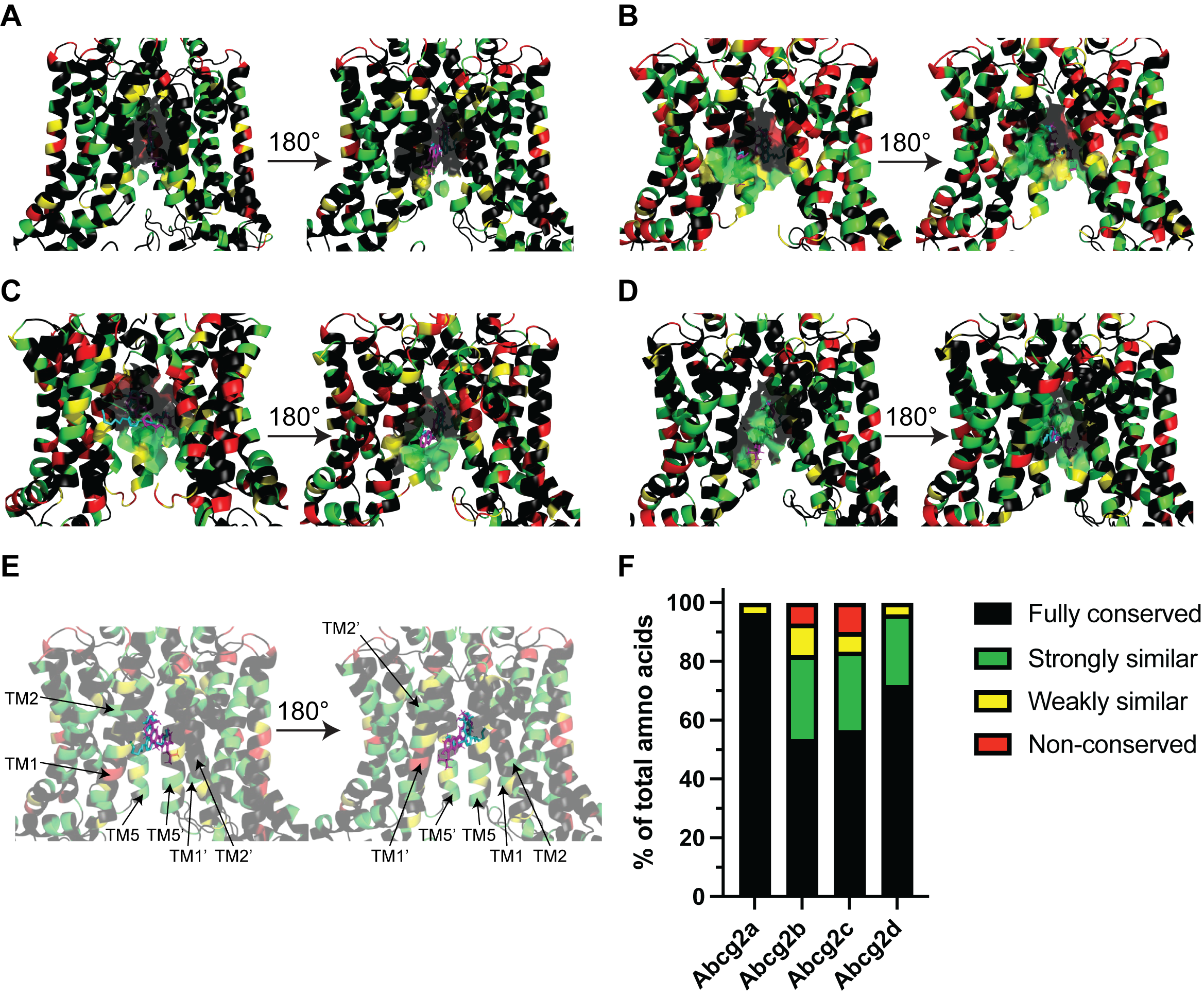

Supplement: Supplementary file 2 — Additional file 2: Figure S2. 3-D homology modeling of amino acid similarity in the substrate binding pocket of A Abcg2a, B Abcg2b, C Abcg2c, and D Abcg2d. The transmembrane region is presented in cartoon mode with a 180° rotated view. Predicted drug-binding pockets, shown in surface mode, focused on residues within a 4.5 Å proximity to the ligands topotecan (magenta) and mitoxantrone (cyan) as identified in human ABCG2 (PDB IDs: 7NEZ and 6VXI, respectively). E A transparent view of panel A with the transmembrane helices labelled from each monomer in the homodimer. F Quantification of the percent of total amino acids in the predicted binding pocket that are fully conserved (black), conservative substitutions (green), semi-conservative substitutions (yellow), or non-conservative substitutions (red), based on similarity to ABCG2. Color coding in the structures A–E is consistent with the graph (F) and Additional file 1: Fig. S1. [file 12987_2024_529_MOESM2_ESM.tif]

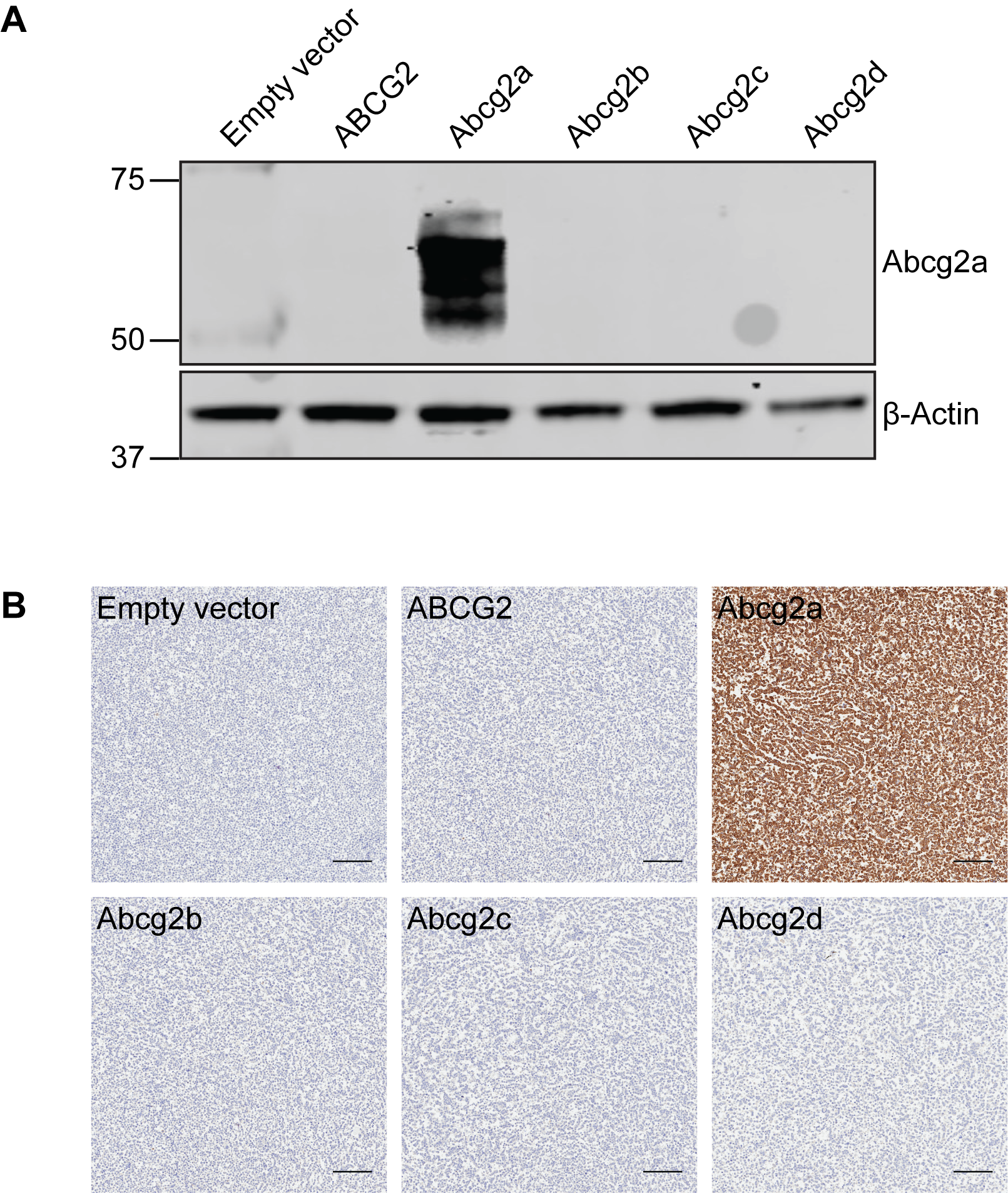

Supplement: Supplementary file 3 — Additional file 3: Figure S3. Abcg2a antibody validation. (A) Immunoblot of total cell lysates and (B) immunohistochemistry of pellets of transfected HEK-293 cells expressing an empty vector, ABCG2, Abcg2a, Abcg2b, Abcg2c, or Abcg2d. Positive signal is only observed in Abcg2a-expressing cells. Scale bar = 100 μm. [file 12987_2024_529_MOESM3_ESM.tif]

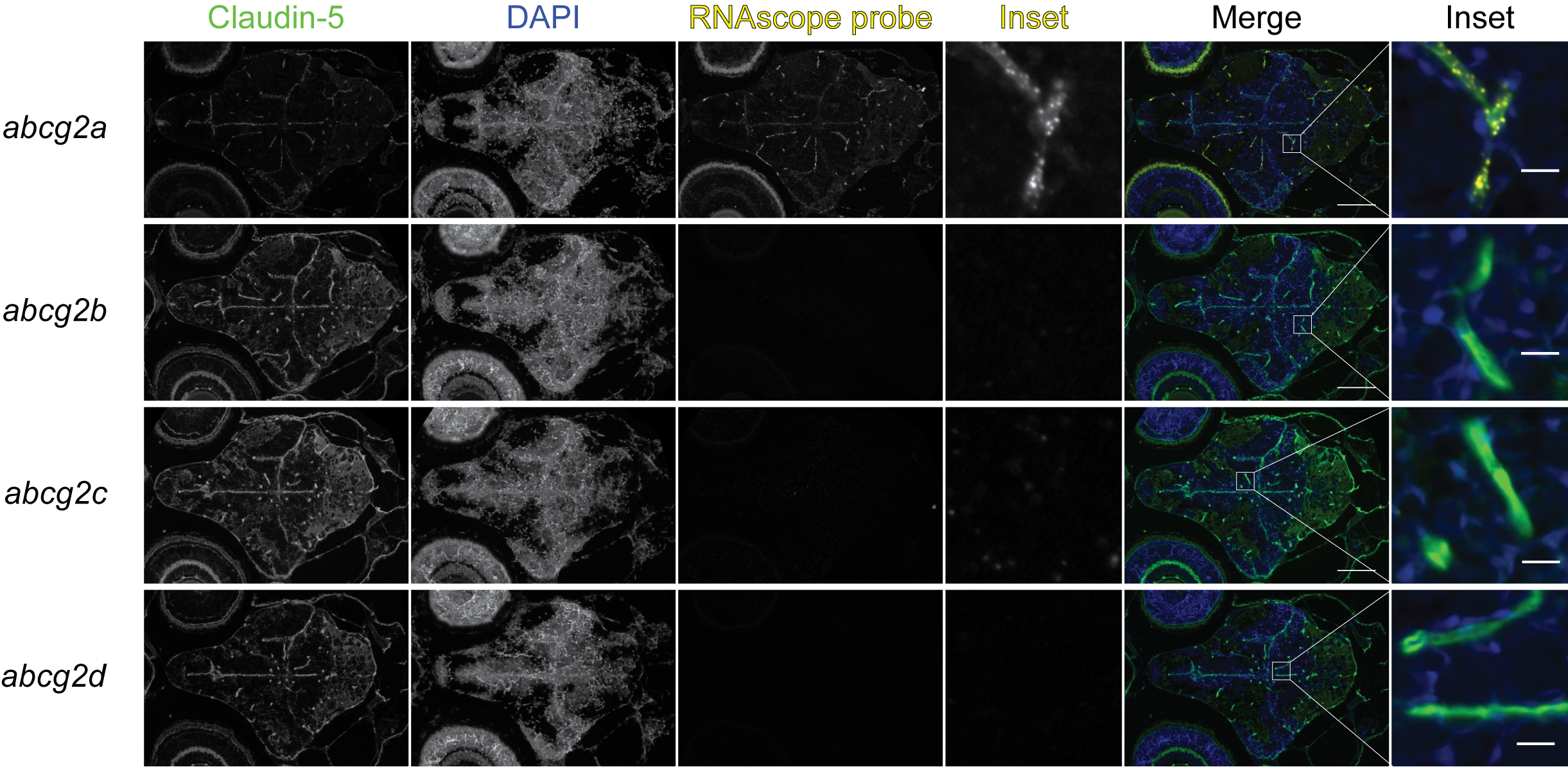

Supplement: Supplementary file 4 — Additional file 4: Figure S4. abcg2a is expressed in claudin-5 positive larval brain vasculature. Paraffin-embedded 7 dpf larval zebrafish sections were probed with RNAscope probes (yellow) to detect abcg2a mRNA, an antibody against claudin-5 (green) and DAPI (blue). Scale bar = 100 μm, inset scale bar = 10 μm. [file 12987_2024_529_MOESM4_ESM.tif]
