# Supplementary material for: Abcg2a is the functional homolog of human ABCG2 expressed at the zebrafish blood–brain barrier
Source: Fluids Barriers CNS. 2024 Mar 15;21:27. doi: 10.1186/s12987-024-00529-5 (PMC10941402; doi:10.1186/s12987-024-00529-5)
Supplement: Supplementary file 5 — Additional file 5: Table S1. Cross-resistance profile of known ABCG2 cytotoxic substrate drugs with zebrafish Abcg2a-d [file 12987_2024_529_MOESM5_ESM.pdf]

Table S1. Cross-resistance profile of known ABCG2 cytotoxic substrate drugs with zebrafish Abcg2a-d[a]

Compound	GI_{50} Empty vector (µM)	GI_{50} ABCG2 (µM)	RR* ABCG2	GI_{50} Abcg2a (µM)	RR* Abcg2a	GI_{50} Abcg2b (µM)	RR* Abcg2b	GI_{50} Abcg2c (µM)	RR* Abcg2c	GI_{50} Abcg2d (µM)	RR* Abcg2d
MLN 7243	0.069±.0050	15.4±.22	223	7.49±.2.8	109	0.151±.051	2.19	0.200±.020	2.90	0.179±.023	2.60
MLN 4924	0.420±.090	7.32±1.7	17.6	2.44±.17	5.86	0.520±.052	1.25	0.487±.040	0.430	0.353±.19	0.850
PF-3758309	0.068±.030	2.36±.54	34.8	1.26±.53	18.5	0.413±.15	6.09	1.97±.71	29.1	1.54±.65	22.7
THZ 531	1.29±.14	65.9±3.7	51.1	18.3±3.6	14.2	10.5±.1.4	8.32	11.4±1.0	8.80	3.99±.24	3.09
CUDC-101	0.780±.10	11.3±2.0	14.5	5.42±1.4	6.96	2.09±.19	2.68	1.69±.29	2.17	1.04±.26	1.33
Gedatolicib	0.200±.010	3.84±1.5	19.1	1.39±.19	6.86	4.06±1.5	20.1	3.26±.49	16.2	0.820±.50	4.07

[a] All compounds were tested in at least 3 biological replicates. Results are mean GI_{50} values with +/- standard deviation.

* Relative resistance (RR) value is the ratio of GI50 values of ABCG2, Abcg2a-d overexpressing cells, to the empty vector
