# Supplementary material for: Abcg2a is the functional homolog of human ABCG2 expressed at the zebrafish blood–brain barrier
Source: Fluids Barriers CNS. 2024 Mar 15;21:27. doi: 10.1186/s12987-024-00529-5 (PMC10941402; doi:10.1186/s12987-024-00529-5)
Supplement: Supplementary file 6 — Additional file 6: Table S2. UniProt percent amino acid identity matrix for human and zebrafish ABCG2 homologs [file 12987_2024_529_MOESM6_ESM.pdf]

Table S2. UniProt percent amino acid identity matrix for human and zebrafish ABCG2 homologs[a]

	Abcg2c	Abcg2b	Abcg2d	Abcg2a	ABCG2
Abcg2c	100	63.05	47.23	46.23	47.61
Abcg2b	63.05	100	48.53	47.86	47.55
Abcg2d	47.23	48.53	100	65.61	57.41
Abc2a	46.23	47.86	65.61	100	61.43
ABCG2	47.61	47.55	57.41	61.43	100

[a]Uniprot accession numbers: Zebrafish Abcg2c, Q08CU5; Abcg2b, Q2Q44; Abcg2d, Q2Q444. Human ABCG2, Q9UNQ0
